# Partition walls as effective protection from bio-aerosols in classrooms – an experimental investigation

**DOI:** 10.3205/dgkh000380

**Published:** 2021-02-26

**Authors:** Philipp Epple, Michael Steppert, Michael Florschütz, Peter Dahlem

**Affiliations:** 1 Coburg University of Applied Sciences, Department of Mechanical Engineering, Coburg, Germany; 2 REGIOMED Medical Center, Coburg, Germany and Faculty of Medicine, University of Split, Croatia

**Keywords:** COVID 19, bio-aerosols, droplets, infection prevention, partition walls, flow visualization, concentration measurements

## Abstract

**Introduction:** During a pandemic, protective measures to prevent bio-aerosol based infections, such as Corona Virus Infection Disease 19 (COVID 19), are very important. Everyday face masks can only partially block aerosols, and their effectiveness also depend on how well the person is wearing it. They are recommended for classroom situations during high pandemic activity. However, ‘unprotected’ communication with and among children is fundamental from the pedagogical and psychological point of view for normal psychosocial development and teaching. Partition walls around the persons can theoretically provide substantial standardized mechanical protection against the spread of droplets and aerosols, either as additional protection to face masks or as an alternative.

**Methods:** In the present research, the protection effectiveness of partition walls was investigated. With mannequin heads, fog generators, line lasers and a classroom-like setup with protective walls, flow visualization and aerosol concentration measurements were performed. Additionally, an active fan-suction system was tested to remove the channelled aerosols on top of the partition walls before they reach other persons in the room.

**Results:** It was found that partition walls protect neighbours from bio-aerosol contact regardless of whether they wear masks or not. The combination with standardized room ventilation enforces this effect. Moreover, the experiments performed here clearly showed that partition walls may protect neighbours from bio-aerosols better than suboptimally fitting everyday face masks only.

**Conclusion:** Partition walls are the most effective protection against infectious bio-aerosols in classroom settings and should be combined with standardized ventilation as the preferred method for classrooms during the current COVID 19 pandemic.

## Zusammenfassung

**Einführung:** Während Pandemien, wie der aktuellen „Corona Virus Infection Disease 19 (COVID 19)“ Pandemie, ist es wichtig, geeignete Schutzmaßnahmen zu etablieren, die eine durch Bioaerosole verursachte Infektion verhindern können. Alltags-Mund-Nasenmasken sind generell für das Tragen in Unterrichtsräumen während einer Pandemie empfohlen. Dennoch können sie die Ausbreitung von Aerosolen nur teilweise verhindern. Ihre Wirksamkeit hängt sehr davon ab, wie korrekt sie getragen werden. Unter pädagogischen und entwicklungspsychologischen Aspekten ist es fundamental wichtig, dass die Kommunikation zwischen Lehrer und Schüler ungehindert unter Einbeziehung der gesamten Mimik des Gesichts stattfinden kann. Trennwände zwischen den im Raum befindlichen Personen könnten theoretisch einen optimalen Schutz vor Tröpfchen und Aerosolen sicherstellen und entweder als Alternative oder Ergänzung zum Mund-Nasen-Schutz angewandt werden.

**Methode:** In der vorliegenden Studie wurde die Effektivität von Trennwänden zum Schutz vor Bioaerosolen untersucht. Mit sogenannten Figurenköpfen, einem Nebelgenerator, einem Linienlaser und einer Sitzanordnung entsprechend einem simulierten Unterrichtsraum wurde mit einerseits Strömungssichtbarmachung und andererseits mit Konzentrationsmessungen der Partikelgrößen die Aerosolausbreitung im Raum untersucht. Mit einer aktiven Absauglüftungsanlage wurde zusätzlich untersucht, wie sich die Aerosole aus dem Unterrichtsraum entfernen lassen, bevor sie in Kontakt mit anderen Personen kommen.

**Resultate:** Die Ergebnisse dieser Untersuchung zeigen, dass Trennwände den direkten Nachbar vor Bioaerosol Kontakt schützen, unabhängig ob Mund-Nasenmasken getragen werden. Die Kombination mit einem Absauglüftungssystem verstärkt diesen positiven Effekt. Zusätzlich zeigen die Messungen, dass Trennwände den direkten Nachbar besser schützen als Alltags-Mund-Nasenmasken.

**Schlussfolgerung:** Trennwände sind die effektivste Schutzmaßnahme gegen infektiöse Bioaerosole in Unterrichtsräumen. In Kombination mit einem Absauglüftungssystem sollten sie als die bevorzugte Schutzmaßnahme während der aktuellen COVID 19 Pandemie angewandt werden.

## What this paper adds

### What is already known about this subject?

- The best prevention of airborne infectious diseases by bio-aerosols is avoiding contacts or establishing mechanical barriers.
- The barrier function of everyday face masks is considered to reach this goal. However, they cannot 100% guarantee spreading of aerosols for different reasons.
- This weakness of face masks is of particular importance in closed classrooms with insufficient air ventilation. Partition walls between people may therefore present a definite barrier for aerosols. However, detailed scientific data are lacking.

### What are the new findings?

- FLOW visualization and aerosol concentration measurements showed that wearing face masks in a school setting will immediately spread aerosols in high concentration to neighbouring persons.
- Further measurements confirmed that partition walls prevented spreading of aerosols to the nearest neighbours, whether face masks are worn or not.

### How might this impact on policy or the foreseeable future?

- Partition walls would allow students to attend their classes and receive their education with or without face masks. The latter would guarantee visible faces, which are essential for appropriate education of children and students.
- School authorities should consider their use in combination with air suctioning systems in the very near future as an optimal setting to prevent classroom-associated bio-aerosol spreading during the ongoing COVID 19 pandemic.

## Introduction

Droplets from the nasopharynx and aerosols spreading in the surroundings may carry infectious agents, such as viruses. This so-called bio-aerosol may infect people by contact and inhalation.

This form of spreading is important during a pandemic such as the present Coronavirus Induced Infectious Disease (COVID) 19 pandemic [1]. In poorly ventilated classrooms, bio-aerosols can spread continuously and pose a high risk of infection for students and teachers [2]. It has been shown that when coughing and sneezing, the aerosols may spread to a distance of 1 m and more [3], [4]. 

This is important in situations where it is not possible to keep a safe distance. Then, mechanical protection such as face masks may be used to block the spread of droplets and aerosols and lower the risk of an infection. Therefore, face masks are the most widely used protective measure to prevent bio-aerosol-based infections such as COVID 19. 

Nevertheless, face masks – especially the everyday face masks – can only partially block the aerosols; their effectiveness also depends on how well the person wears it [5], [6]. In the case of children in schools, the percentage of face masks that are not worn properly is much higher than in the case of adults, and hence, the protection offered by the face mask is reduced. This is even more important due to the fact that the topographic anatomy of a face is very individual, and in children, the face is smaller. In classrooms, where no face masks are worn, there is no protection against droplets and aerosols. However, in classroom situations, unprotected communication with and among the children is fundamental from pedagogical and psychological points of view for normal psychosocial development and teaching [7]. 

Partition walls around the persons can theoretically provide substantial, standardized mechanical protection against the spread of droplets and aerosols, either as additional protection to face masks or as an alternative means [8].

To the knowledge of the authors, no detailed, systematic scientific investigation of the protection effectiveness of partition walls has been published to date.

In the present study, the protection effectiveness of partition walls in a simulated classroom setting was investigated systematically by flow visualization methods and aerosol concentration measurements. 

## Methods

General flow visualization methods are described in the experimental fluid mechanics literature, for example, in Smits and Lim [9] and Nitsche [10]. However, each experimental setup has its own special characteristics that must be considered. In the case of the classroom setup. it was necessary to choose a flow visualization method that covers a larger area. As an example, Stymates [11] used a schlieren imaging and light-scattering method. This method visualizes density variations and can thus show a person’s exhaled air, which is less dense due to its higher temperature. It has the advantage that no fog is needed, but it is restricted to small areas due to the required optics, and the resulting images are in black and white. For the present study, a large visualization area was needed to show the aerosols in a classroom; hence, a schlieren method is not suitable. Verma [12] used a laser sheet generated by a line laser and tracers composed of droplets of distilled water and glycerin to visualize the exhalation of a mannequin head. They used a pump to exhale the droplets of distilled water and glycerin. Basically, this is also the method chosen for this research. However, instead of the pump, a continuous fog generator was used to simulate the continuous exhalation of aerosols over longer periods of time, simulating a realistic scenario in a classroom. Therefore, for the flow visualization of aerosols in classroom setups with partition walls, a combination of flow visualization with fog and line lasers was chosen because it clearly visualizes the flow with high contrast over a long period of time. With this method of continuous fog generation combined with line lasers, the flow in a large area of the classroom could also be visualized. Aerosol concentration sensors were used for the additional quantitative aerosol concentration measurements as a function of time. 

**Figure 1:**
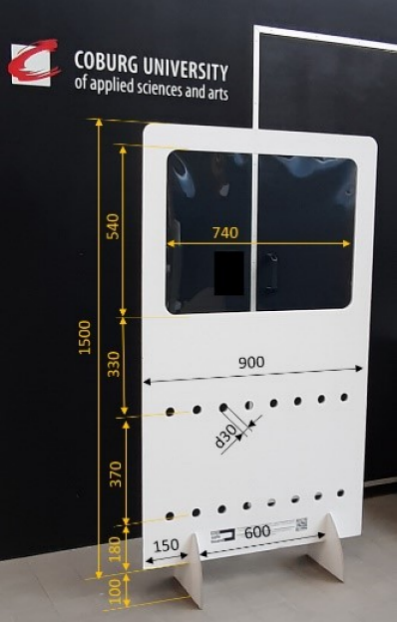
Partition wall of corrugated cardboard with a plastic foil window

**Figure 2:**
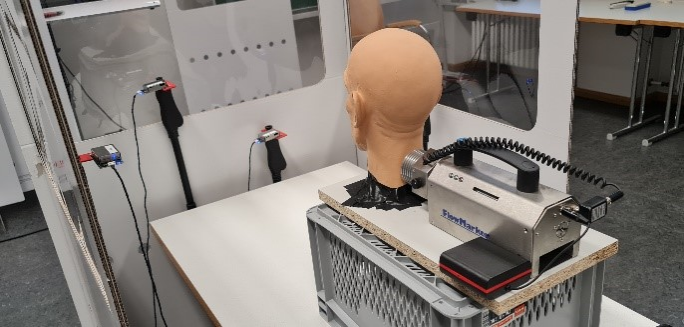
Figure 2: Mannequin head, FlowMarker^®^, particle sensors Plantower PMS 7003 and partition walls setup for reference values

The whole experimental setup consisted of partition walls of corrugated cardboard (Schumacher Pack Solution GmbH, Figure 1 (Fig. 1)), and two fog generators (FlowMarker^®^ and FlowLiner^®^ from Tintschl Bioenergie und Strömungstechnik Ag). The FlowMarker^®^ (Figure 2 (Fig. 2)) generates fog at lower velocities than FlowLiner^®^. The fog liquid used was Safex Flowmarker. To measure the aerosol concentrations, four Plantower PMS 7003 sensors were used (Figure 2 (Fig. 2)). These sensors measure the particle classes PM1.0, PM2.5 and PM10, and the number of particles of sizes 0.3 µm, 0.5 µm, 1.0 µm, 2.5 µm, 5.0 µm and 10.0 µm in 100 ml of air could be determined. 

The setup to measure the reference values is shown in Figure 2 (Fig. 2). 

This setup was used to determine the aerosol concentration expelled by the FlowMarker^®^ through the mannequin head and which remains close to the mannequin head in the space enclosed by the three partition walls, i.e., in the surroundings of the mannequin head when it is enclosed by the partition walls. 

In addition to the fog generator, a class-2 line laser with 15 mW of power and a wavelength of λ=532 nm (green) was used. Finally, a fan with a flow rate of Q=410 m³/h and an Alu-Flex pipe was used to suck the aerosols out of the top of the partition wall cabins. 

### Flow visualization and aerosol concentration measurements

**Figure 3:**
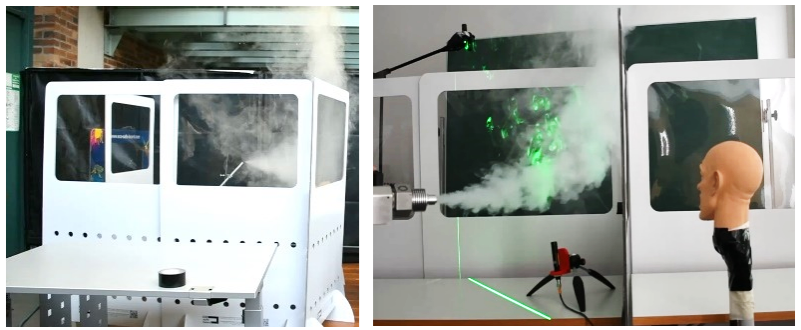
Direct fog application on partition walls with the FlowLiner^®^ (left) and the FlowMarker^®^ (right)

To visually investigate the protective effectiveness of partition walls against the spreading of aerosols. fog was applied with the FlowLiner® and the FlowMarker^®^ directly onto the protective walls as shown in Figure 3 (Fig. 3).

A systematic series of experiments was performed to investigate the effectiveness of face masks and partition walls and to compare them. Two types of experiments were performed: a) flow visualization experiments with fog and in many cases also using the laser to enhance the visualization; b) aerosol concentration measurement experiments, where the aerosols measured came from the fog generator. 

**a)** **Flow visualization**

1.
  1.
    1. Effectiveness of face masks
  2.
    1. Protection effectiveness of partition walls against direct fog impact
  3.
    1. Effectiveness of fan suction system
  4.
    1. Protection effectiveness of partition walls in classrooms without face masks
  5.
    1. Protection effectiveness of face masks in classrooms

**b)** **Aerosol concentration measurements**

1.
  1.
    1. Reference concentration measurement
  2.
    1. Aerosol concentration measurements in classrooms with partition walls

The flow visualization experiments were analysed qualitatively and visually through pictures. The results of the aerosol concentration measurements are presented in x–y charts in the following sections. 

**Figure 4:**
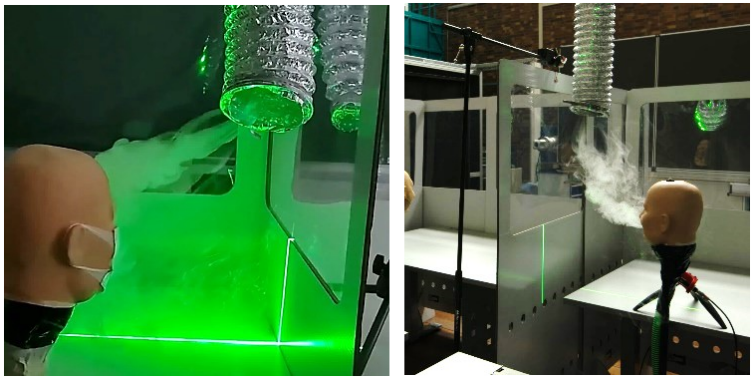
Fan suction system

The effectiveness of a simple suction system consisting of an axial fan and an Alu-Flex pipe was investigated qualitatively with flow visualization (Figure 4 (Fig. 4)).

## Results

**Figure 5:**
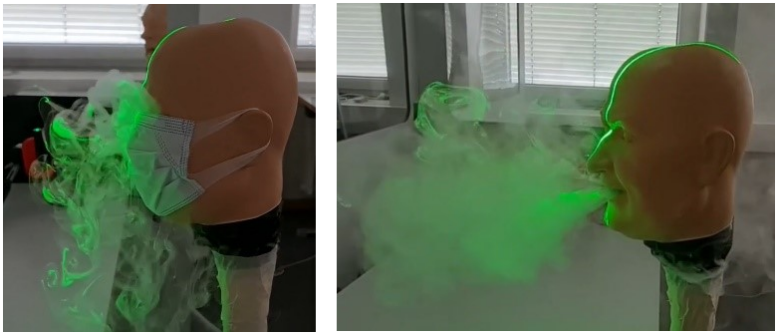
Flow visualization with and without face mask

In this part of the study, the flow with and without an everyday face mask was visualized with fog and a green line laser, as shown in Figure 5 (Fig. 5).

**Figure 6:**
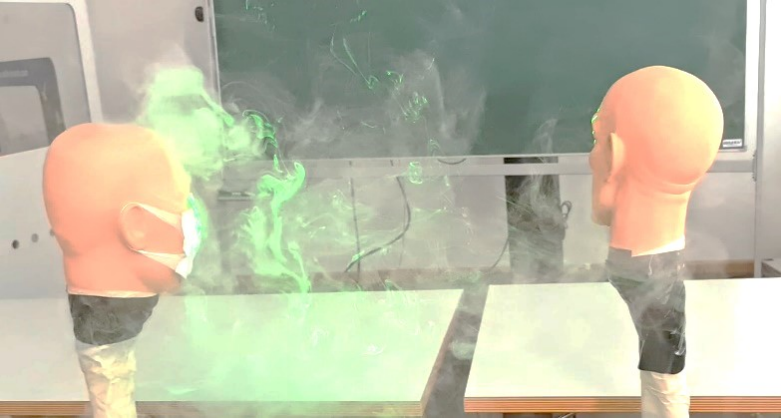
Even with everyday face masks, the aerosols spread into the room and reach the neighbour.

Besides the filtering effect of the face mask, the flow velocity of the expelled aerosols and droplets is also reduced compared to an uncovered face. However, we clearly demonstrated that a large amount of aerosol still leaks along the mask and enters the classroom, easily reaching neighbouring persons (Figure 6 (Fig. 6)).

In contrast, the flow visualizations in Figure 3 (Fig. 3) show that the partition walls prevent the fog from passing to the other side of the wall. The droplets and aerosol do not reach the neighbour, who is immediately protected even during high velocity output during coughing and sneezing.

**Figure 7:**
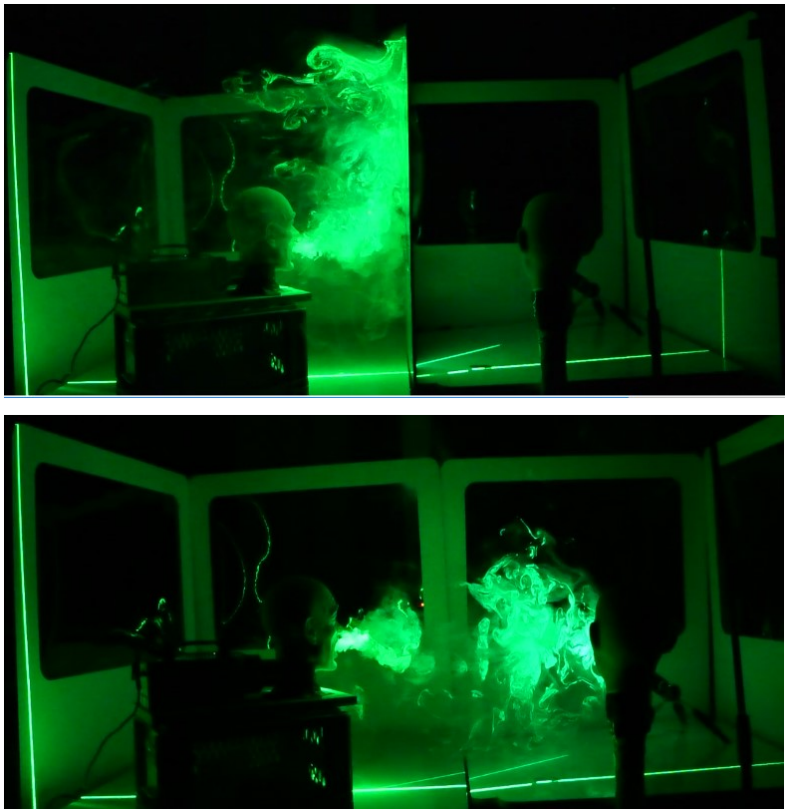
Protective effectiveness of partition walls: (above) the partition wall prevents the mannequin head on the right from the aerosols; (below) without partition walls the aerosols reach the right-hand mannequin head after a short time.

The effectiveness of a simple suction system consisting of an axial fan and an Alu-Flex pipe is depicted in Figure 4 (Fig. 4). The experiments showed that the aerosols tend to flow upwards, stay in the cabin and accumulate on top of them. From there, the expelled aerosol is sucked off effectively with the fan. Neighbours are not contaminated (Figure 7 (Fig. 7)).

**Figure 8:**
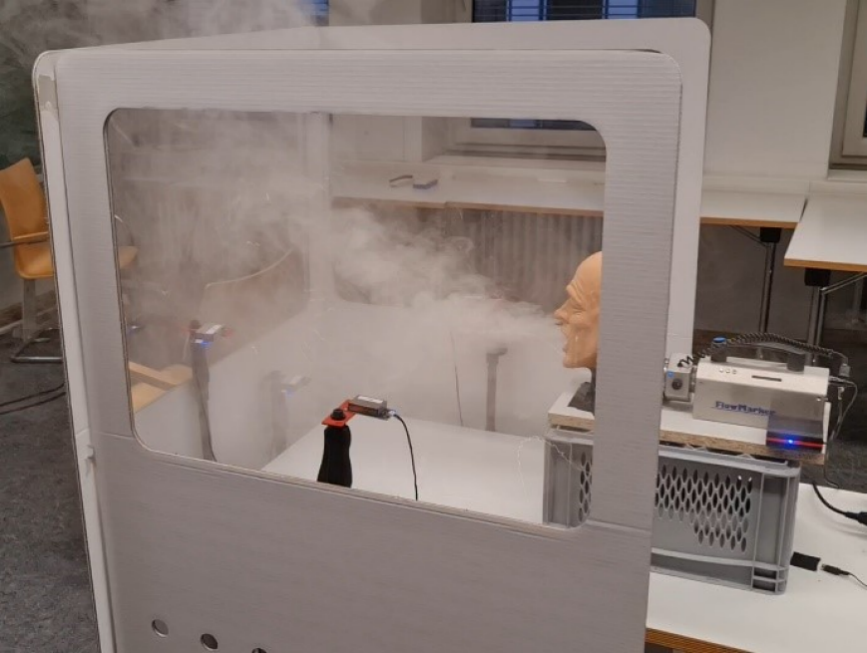
Reference using three-partition wall cabin with FlowMarker^®^ and four Plantower PMS 7003 sensors

After the qualitative flow visualization experiments, quantitative experiments were performed to measure the aerosol concentration in different situations. Before starting with the comparative aerosol concentration measurements, a reference measurement in a single three-partition wall cabin was performed (Figure 8 (Fig. 8)).

**Figure 9:**
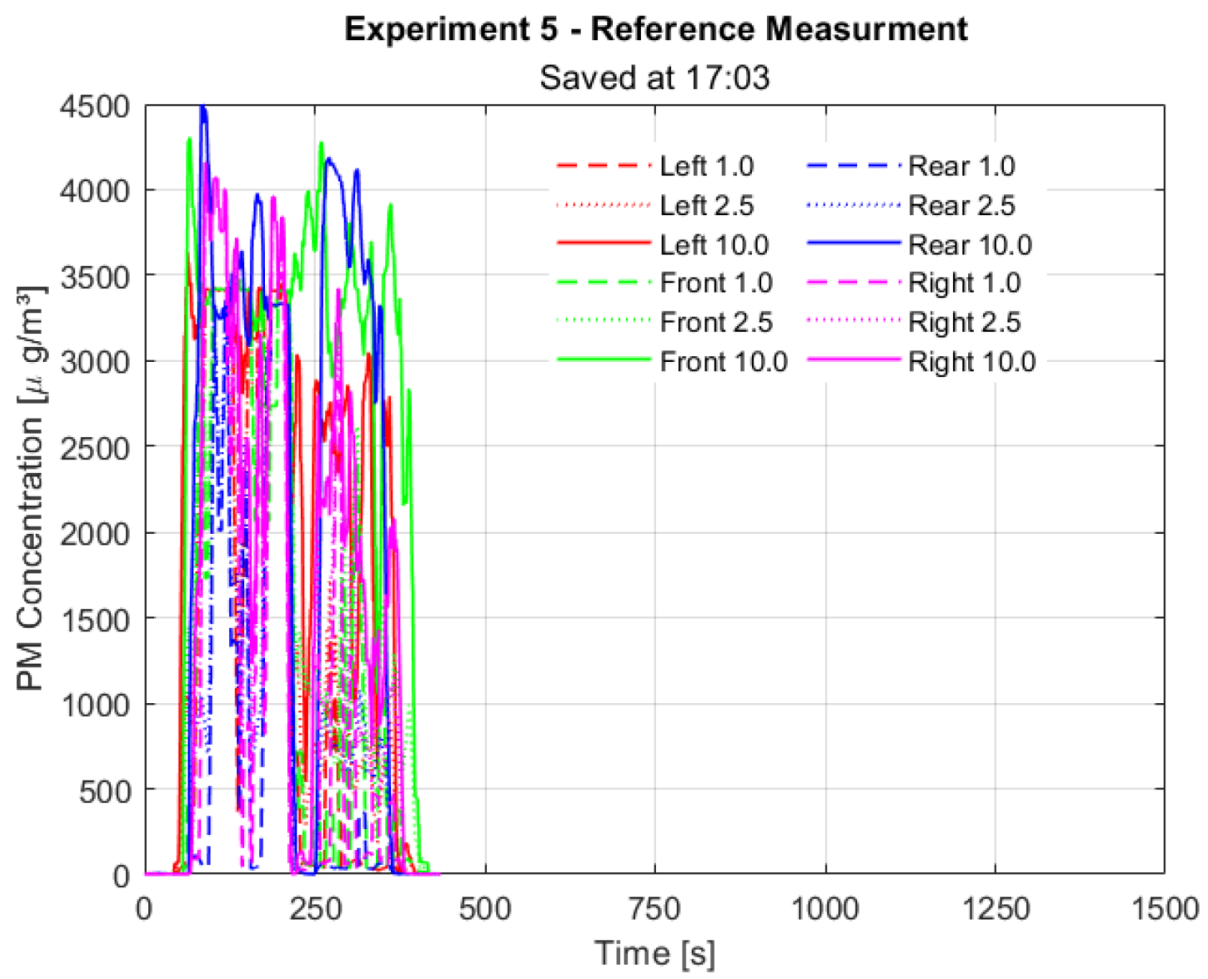
Reference measurements in the cabin.

The results of the reference measurements are shown in Figure 9 (Fig. 9). The maximum aerosol concentration measured was about 4.000 µg/m^3^.

**Figure 10:**
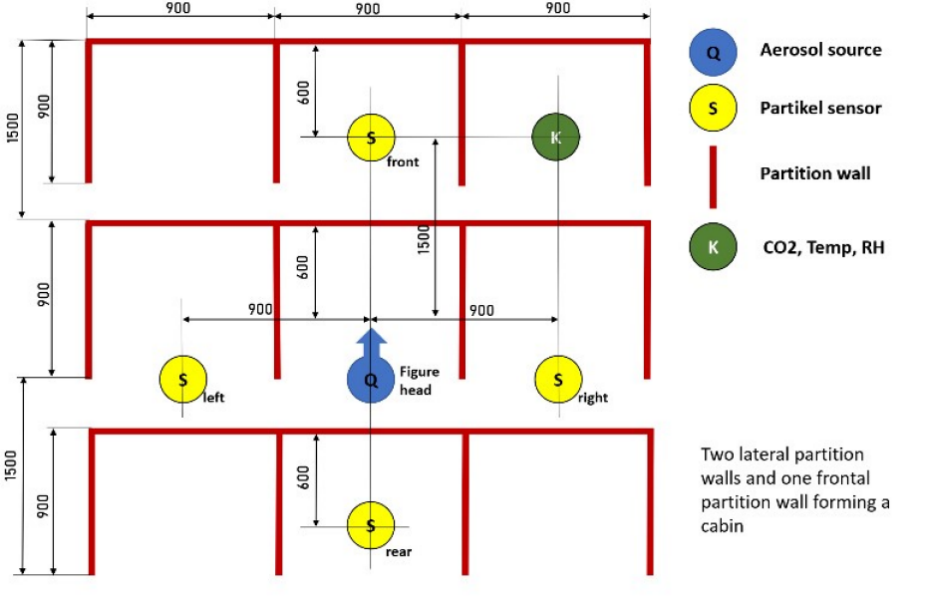
Classroom setup

To quantify the effectiveness of partition walls against the spreading of aerosols, measurements were made in a classroom setup, as shown in Figure 10 (Fig. 10). The mannequin head with the aerosol source (FlowMarker^®^) were placed on the central table. Particle sensors (S) were placed at the height of the mannequin head on the tables in front, behind, left and right of the mannequin head with the aerosol source (Q). With these sensors, the particle concentrations were measured as a function of time. In the front on the right side, another sensor (K) was also placed to measure the CO_2_ concentration, the ambient temperature and the relative humidity. The camera was placed on the left side of the back left table. 

**Figure 11:**
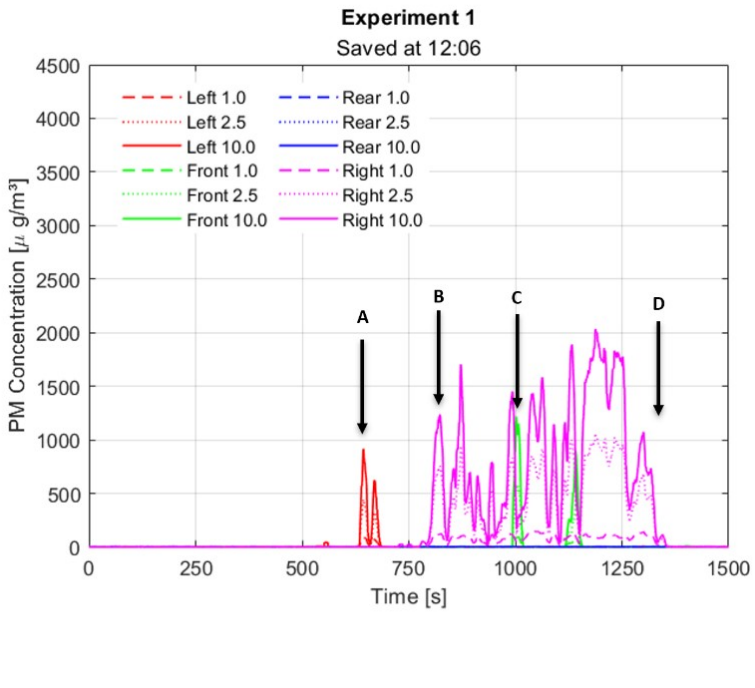
Aerosol concentration at the four sensors

The results of the measurements are shown in Figure 11 (Fig. 11). It can be seen from the graphs that up to about 600 seconds, i.e., 10 minutes, none of the sensors registered any substantial aerosols. That is, the aerosols expelled by the figure head (Q; Figure 10 (Fig. 10)) do not reach the sensors (S) since the partition walls are between them.

**Figure 12:**
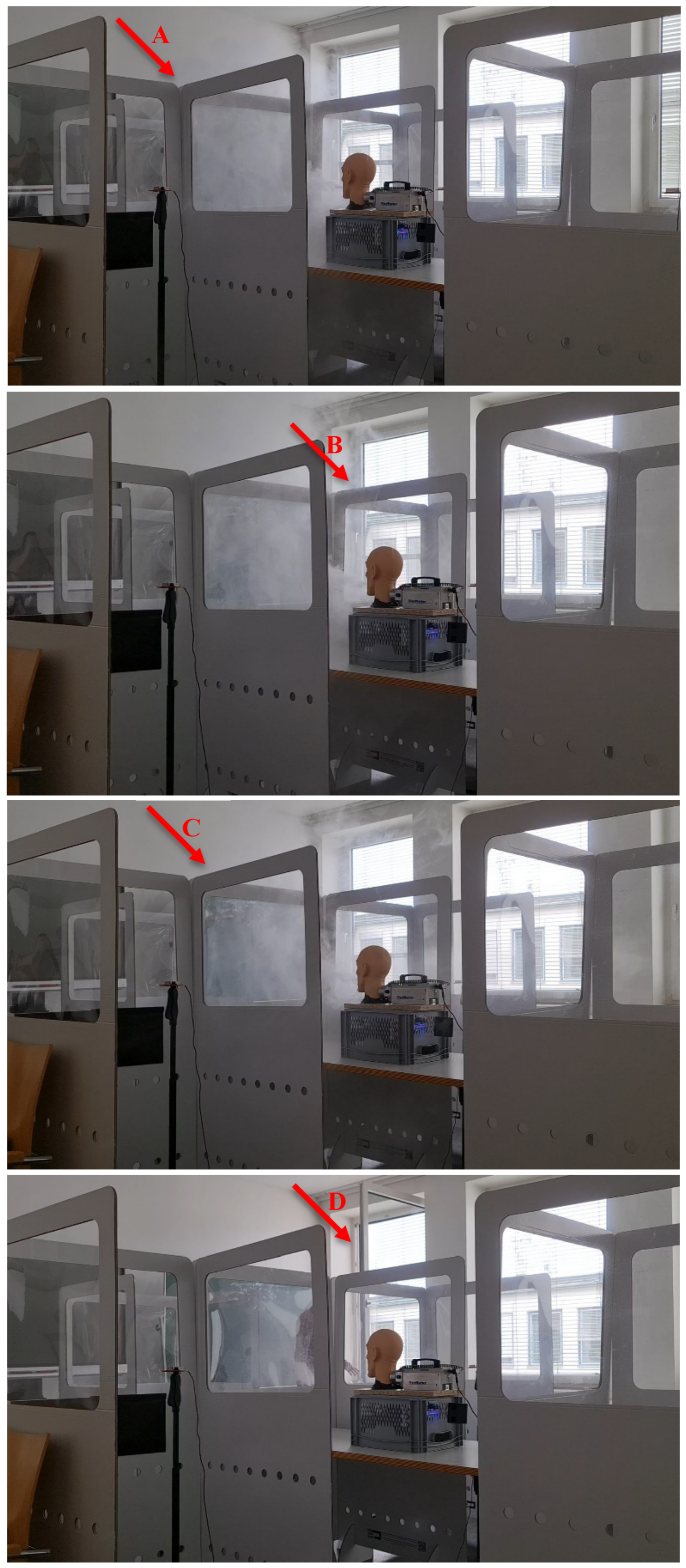
Measurement of aerosols with partition walls, situations A, B, C and D

From about 600 s on, four main events were observed and are also shown in Figure 12 (Fig. 12). As compared with the reference values of Figure 9 (Fig. 9), one can see that, although from about 600 s there is an aerosol concentration detected in some of the neighbouring cabins, these values are all less than half of the reference values of about 4.000 µg/m^3^. Hence, even if some aerosol flows over to the neighbouring cabin, its concentration is much lower than the reference values. 

The four stages A, B, C and D of the experiment can be described as follows:

- A) At about 10 min, some of the fog reaches the left sensor, flowing over the left partition wall, as shown by the arrow A in Figure 12 (Fig. 12).
- B) At about 800 s, i.e., about 13.3 min, the right sensor started to show an elevated aerosol concentration. Here, fog flows over the right partition wall (arrow B in Figure 12 (Fig. 12)) and reaches the right-hand cabin and the sensor inside it.
- C) At about 1,000 s, i.e., about 17 min, the front sensor shows an elevated aerosol concentration. Here, the fog from the central cabin flows over the front wall and reaches the front cabin and sensor (arrow C, Figure 12 (Fig. 12)).
- D) At about 1,200 s, i.e., about 20 min, the mannequin head stopped producing fog, i.e., aerosols, and a window was opened for ventilation (arrow D, Figure 12 (Fig. 12)). After stopping the emission of aerosols and opening the window, the aerosol concentration dropped to zero again within a few minutes, showing how efficient ventilation was in this case.

With a suction system on top of the cabins, the overflow of aerosols from the central to the right cabin to a neighbouring cabin could probably be avoided, and the aerosols generated in the central cabin could be removed directly from the room.

The cost of installation of the partition walls and an industrial air fan-suction system including fans, extractor hoods, and flue pipes in a classroom for about 20 pupils was about 3,500 Euro.

## Discussion of the results

With this series of flow visualization experiments and aerosol concentration measurements, it was possible to show details of aerosol spread in closed rooms, such as classrooms, and of the protective effectiveness of partition walls.

Firstly, we again demonstrated that the effectiveness of face masks is limited under certain circumstances. Seminara et al. [4] and Dbouk [5] analyzed the protective limits of surgical masks. Verma et al. [12] also performed visualization using fog and a green line laser, but they investigated face shields and masks with exhalation valves. They concluded that their effectiveness was relatively low. Everyday face masks provide only limited protection, since aerosols with particle size smaller than droplets will leak through the gaps between face and mask (Figure 5 (Fig. 5) and Figure 6 (Fig. 6)). Only recently has this disadvantage of everyday face masks been demonstrated clearly, and might explain why SARS CoV 2 is still spreading [6].

Therefore, persons remaining in a room for a longer period will in the long run inhale aerosols from their neighbours. Moreover, in a classroom situation, the students sit quite close together, as shown in our simulation with the mannequin heads (Figure 6 (Fig. 6)), and the aerosols reach the neighbours in a short time despite wearing face masks. 

Secondly, we for the first time clearly showed the protective effect of partition walls in a simulated classroom setting. In the setting of our flow experiments, the protection from aerosol is even better than with face masks only, since, as described above, aerosols escape from masks, but not from the cabin. 

The quantitative experiments with the particle sensors additionally showed that compared to the reference aerosol concentration generated in one cabin, the aerosol concentration in its neighbouring cabins was always below of half of the reference. During a long period of at least 10 minutes, these values even approached zero before the aerosol was further spread into the classroom. Installing a fan suction system, the aerosols can be removed directly from the top of the partition wall cabins and all neighbours will be protected.

In a preliminary setup, we could demonstrate the beneficial effect of eliminating the expired aerosol directly by a simple suctioning system (Figure 4 (Fig. 4)). Hence, it is proposed that a suction system should be used in combination with partition walls in classrooms to continuously collect and remove the aerosols from the room. The exact design of the suction system was not part of this investigations and must be developed in a separate work. This also needs to be defined for a standardized ventilation protocol through opening the windows.

Of course, partition walls with transparent plastic foil windows offer the great advantage of enabling visual communication between the teachers, students and pupils, which is otherwise substantially impaired by covering the faces with masks. Keeping pupils communicating during lessons is of paramount importance for their scholastic progress as well as mental and emotional development [7]. This is essential for communication and learning, in addition to prevention of the negative psychological effects of face masks [13], [14].

These partition walls would allow lessons without face masks even during a pandemic period.

The limitation of this study is the fact that the complete setting with the dimensions of the partition walls and with suction system is only preliminary, with the intent to investigate the fundamental protective impact of partition walls. Therefore, the exact dimensions of the partition walls, setting and suction system remain to be optimized and adjusted to ensure a safe and optimal operation considering all aspects of a classroom. This will be a prerequisite, for instance, for obtaining official approval and certification for safe operation in classrooms during a pandemic. 

## Conclusions and outlook

Partition walls are even more effective than everyday face masks against bio-aerosol spreading in classroom settings. Their implementation in classrooms is therefore recommended. Further studies should focus on the optimal material, size and position of the partition walls in combination with a standardized air suctioning or ventilation system.

## Notes

### Competing interests

The authors declare that they have no competing interests.
